# New Insights into PI3K Inhibitor Design using X-ray Structures of PI3Kα Complexed with a Potent Lead Compound

**DOI:** 10.1038/s41598-017-15260-5

**Published:** 2017-11-06

**Authors:** Xiuyan Yang, Xi Zhang, Min Huang, Kun Song, Xuefen Li, Meilang Huang, Linghua Meng, Jian Zhang

**Affiliations:** 1 Institute of Bioinformatics and Medical Engineering, School of Electrical and Information Engineering, Jiangsu University of Technology, Changzhou, 213001 China; 2 Department of Pathophysiology, Chemical Biology Division of Shanghai Universities E-Institutes, Key Laboratory of Cell Differentiation and Apoptosis of Chinese Ministry of Education, Shanghai JiaoTong University, School of Medicine, Shanghai, 200025 China; 3 Division of Anti-tumor Pharmacology, State Key Laboratory of Drug Research, Shanghai Institute of Materia Medica, Chinese Academy of Sciences, Shanghai, 201203 China; 4 School of Chemistry and Chemical Engineering, Queen’s University Belfast, Northern Ireland, United Kingdom; 5 Department of Hepatobiliary Surgery, Xijing Hospital, Fourth Military Medical University,, Xi’an, China

## Abstract

Phosphatidylinositol 3-kinase α is an attractive target to potentially treat a range of cancers. Herein, we described the evolution of a reported PI3K inhibitor into a moderate PI3Kα inhibitor with a low molecular weight. We used X-ray crystallography to describe the accurate binding mode of the compound YXY-4F. A comparison of the p110α–YXY-4F and *apo* p110α complexes showed that YXY-4F induced additional space by promoting a flexible conformational change in residues Ser773 and Ser774 in the PI3Kα ATP catalytic site. Specifically, residue 773(S) in PI3Kα is quite different from that of PI3Kβ (D), γ (A), and δ (D), which might guide further optimization of substituents around the NH group and phenyl group to improve the selectivity and potency of PI3Kα.

## Introduction

Phosphatidylinositol 3-kinases (PI3Ks) are lipid kinases that play pivotal roles in a multitude of fundamental biological processes, including proliferation, survival, differentiation, and metabolism^1–5^. Recent reports have shown that the PI3K/AKT/mTOR pathway is crucial in T cell activation and function^6^. PI3Ks are currently divided into classes IA, IB, II, and III^7^. The class IA family consists of three isoforms, α, β, and δ^8^. The class IB family consists of only the γ subtype. Among the different PI3K subfamily proteins, PI3Kα is the most important isoform in cell proliferation in response to growth factor-tyrosine kinase pathway activation^9,10^. PI3Kα is a heterodimer that contains a p110α catalytic subunit and p85α regulatory subunit^11–13^. The gene encoding the p110α subunit, *PIK3CA*, is mutated at a rate of nearly 30% in human cancers, including colorectal cancer, glioblastoma, and gastric cancer. Importantly, hyper-activation of PI3Kα directly correlates with resistance to current therapeutic agents and poor prognosis in most human cancers^14–16^. Therefore, PI3Kα is a potential target for anti-cancer drug development.

Currently, there are a number of PI3K inhibitors in clinical development, including Buparlisib (Trial phase III), Pictilisib (Trial phase II), PX-866 (Trial phase II), Pilaralisib (Trial phase II), and Copanlisib (Trial phase I/II)^17–26^. In particular, the first isoform-specific PI3K inhibitor, idelalisib, a selective δ-isoform inhibitor, has been approved in the USA and Europe for the treatment of chronic lymphocytic leukemia, follicular B-cell non-Hodgkin lymphoma, and small lymphocytic lymphoma; idelalisib will provide valuable information and references on PI3Kα inhibitors as drug candidates^27^. The PI3Kα inhibitor with the most potential, Alpelisib, is in a Phase III randomized trial in patients with HR+/HER2–advanced breast cancers^28–31^. Additionally, MLN1117, a potent selective PI3Kα inhibitor, is in a Phase II trial in patients with advanced solid tumors^32,33^.

The design of new potent PI3Kα inhibitors with drug-like properties is still a major challenge because of the highly conserved lipid kinase ATP binding sites and the similarity between their three-dimensional structures. Currently, X-ray crystallography is one of the most useful experimental methods employed in structure-assisted drug design. Co-crystal structures of the protein of interest and ligands provide accurate structural insights into binding sites. Several complex structures of PI3Kα and its ligands have been reported; however, limited useful information can be extracted from the existing structures^12,13,34,35^. Here, we determined the crystal structure of PI3Kα in complex with the potential inhibitor YXY-4F. The description of the new crystal structure provides further insights into the potent and selective design of PI3Kα inhibitors.

## Results

### Identification of YXY-4F as a potent PI3Kα inhibitor

**Figure 1:**
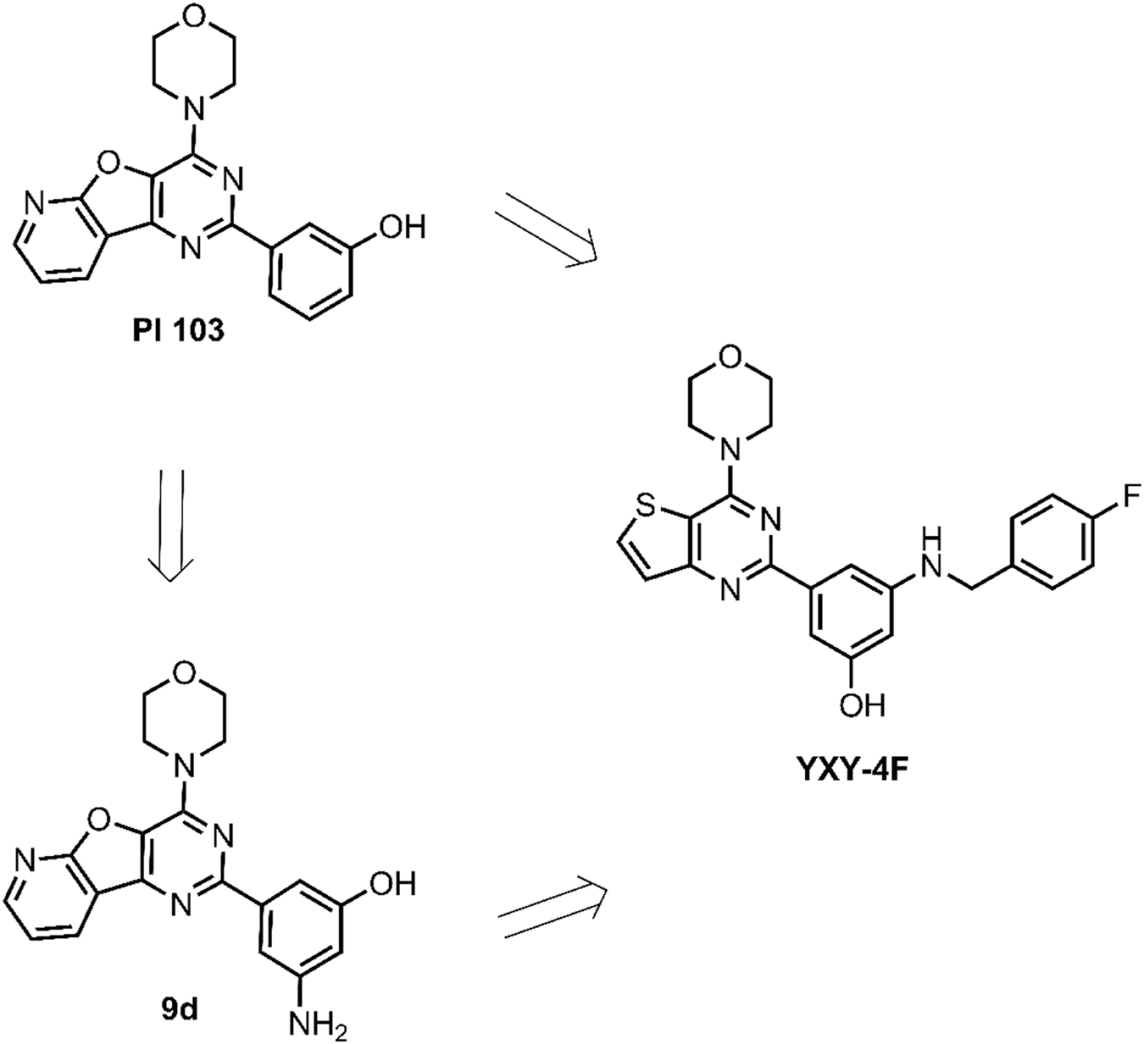
Structure of Thieno[3,2-d]pyrimidine Derivative YXY-4F, 9d and PI 103.

In our previous research^36^, we designed a series of PI-103 derivatives and confirmed that compound **9e** is a potent analogue that can be used for further optimization. PI3Kα-9d crystal-guided optimization led to the discovery of the thieno[3,2-d]pyrimidine derivative YXY-4F (Fig. 1), which is a potent PI3Kα inhibitor with moderate inhibitory activity. As measured by the PI3-Kinase HTRF assay, the compound had an IC_50_ value of 1.02 ± 0.005 μM for the inhibition of p110α. To investigate the cellular effects of our compound, we performed a proliferation assay in *PIK3CA*-driven cell lines (SKOV3, PC-3, and Rh30) treated with the compound. The growth of the *PIK3CA*-driven cell lines was inhibited by exposure to YXY-4F. These data suggest that YXY-4F has a valid anti-proliferative effect in all PIK3CA-driven cancer cells (Table 1). YXY-4F is approximately 50 times less potent than PI-103 in the PI3-Kinase HTRF assay, while in cell inhibitory activity test, YXY-4F is only 10 times less potent than PI-103. Compared to PI-103, YXY-4F has a lower molecular weight and the potential to improve its inhibitory activity by further optimization. Considering these profiles, we chose YXY-4F as the starting point for our medicinal chemistry research. Therefore, the co-crystal structure of PI3Kα and YXY-4F is crucial for crystal-based selective design of PI3Kα inhibitors.

**Table 1:** The test compound YXY-4F inhibit proliferation of cancer cella.

	GI_50_(µM)^b^		
	PC-3	Rh30	SKOV3
PI-103	0.449 ± 0.295	0.549 ± 0.312	0.273 ± 0.177
YXY-4F	3.800 ± 0.291	4.570 ± 0.095	2.436 ± 0.312

^a^Cell proliferation was assessed by an SRB assay as described in the Experimental Section. ^b^GI_50_ values shown are the average ± SD of at least three independent experiments performed in triplicate.

### Crystallization of p110α complexed with YXY-4F

**Figure 2:**
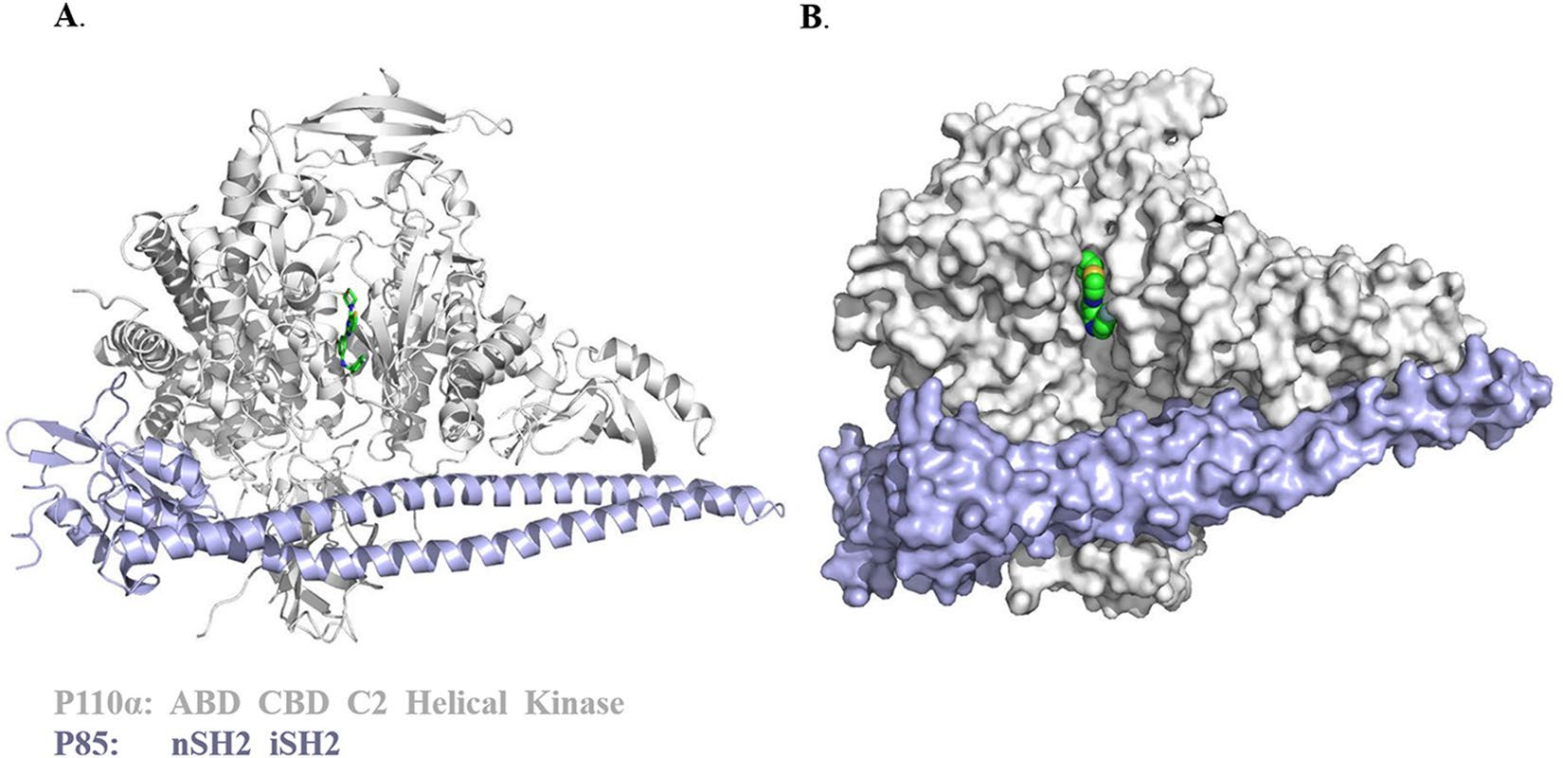
Overview of the p110α/niSH2 heterodimer. (**A**) Diagram of the p110α/niSH2 heterodimer. The compound YXY-4F bound in the kinase domain is shown as sticks. (**B**) Surface diagram of the p110α/niSH2 heterodimer, alternate view. The compound YXY-4F bound in the kinase domain is shown as spheres.

To obtain the co-crystal structure of PI3Kα and YXY-4F, we prepared and purified the protein for X-ray crystallography studies. The fused human PI3Kα gene template containing p110α and p85 linked by a sequence was sub-cloned into the vector. After transposition into the bacmid, a selected positive clone was transfected into SF9 cells to express the protein for co-crystallization with YXY-4F. The harvested protein was purified for crystallization. After screening many crystallization conditions, a complex co-crystal was identified in a solution containing 0.2 M lithium sulfate, 0.1 M Tris pH 8.5, 10 mM YXY-4F, and PEG at a 5:1 ratio at 18 °C. X-ray characterization and data collection for the co-complex were performed, and the diffraction data were processed using *HKL2000*. The data processing and refinement statistics are summarized in Table 2. The final resolution was 2.97 Å, and the R_work_/R_free_ value was 23.1/28.0. The structure maintained all five p110α domains and a long p85 SH2 domain in an overall triangular shape (Fig. 2A).

**Table 2:** Data collection and Refinement Statistic of the Crystal Structure.

PDB ID code	PI3K/YXY-4F, 5XGH		
**(A) Data Collection**			
Resolution (Å)*	50–3.00 (3.11–3.00)	γ (°)	90.00
Space group	P212121	Total reflections	196862
Cell dimensions		Unique reflections	30157
a (Å)	70.360	Completeness (%)*	100.0 (100.0)
b (Å)	136.312	Multiplicity*	6.5 (6.2)
c (Å)	149.408	Average I/σ(I)*	11.5 (2.0)
α (°)	90.00	Rmerge (%)*	14.4 (82.4)
β (°)	90.00		
(**B**) **Refinement**			
Rwork (%)	23.1	Average B Value (Å²)	88.752
Rfree (%)	28.0	Protein Mean B Value (Å²)	88.886
RMSD in Bond Lengths (Å)	0.007	Ligand Mean B Value (Å²)	67.189
RMSD in Bond Angles (°)	0.959	Water Mean B Value (Å²)	48.606
Number of Atoms		Ramachandran Statistics	
Total	10540	Most favored regions	91.4%
Protein	10480	Additional allowed regions	8.6%
Ligand	31	Generously allowed regions	0.0%
Water	18	Disallowed regions	0.0%
B factor Statistics			

^*^Values in parentheses are for highest-resolution shell.

**Figure 3:**
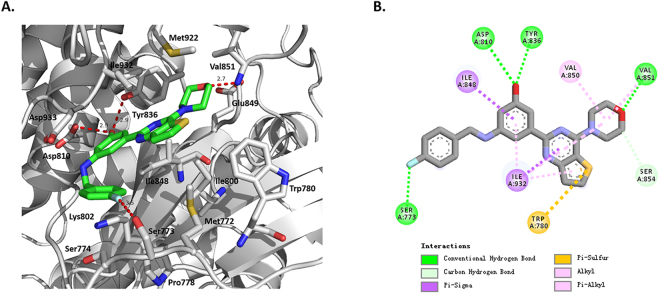
The interactions between YXY-4F and p110α. (**A**) X-ray complex of YXY-4F to p110α (PDB ID: 5XGH). The structure of YXY-4F is shown as a stick representation, and the key binding site residues are shown as sticks. Hydrogen bonds between YXY-4F and the protein are shown as a dashed line Diagram of the p110α/niSH2 heterodimer. (**B**) Extensive residue–residue interactions on the interface of the YXY-4F-p110α complex structure. The residues belonging to p110α are labeled in ball. A distance between donor and acceptor of less than 3.5 Å indicates a hydrogen bond, and a 4.1 Å distance between two hydrophobic atoms indicates a hydrophobic interaction.

We next described the inhibitor binding mode. The crystal structure of PI3Kα–YXY-4F verified that the compound YXY-4F bound to the ATP-binding pocket of the p110α kinase catalytic domain (Fig. 2B). YXY-4F was anchored by multiple hydrogen bonds and hydrophobic interactions within the pocket formed by residues Ile800, Asp810, Tyr836, Glu849, and Val851 on one side and residues Met922, Ile932, and Asp933 on the other side (Fig. 3A). Four H-bonds were formed between YXY-4F and the p110α active site residues. The morpholino oxygen accepted a hydrogen bond from the amide of the hinge Val851 residue. The fluorine in the phenyl moiety also accepted a hydrogen bond from the Ser773 hydroxyl group. The hydroxyl group in the phenol moiety formed two H-bonds with the Asp810 carboxyl side chain and Tyr836 hydroxyl group (Fig. 3B). Additionally, van der Waals interactions from the surrounding hydrophobic residues, including Val850, Ile932, Ile848 and Pro778, significantly contributed to YXY-4F’s affinity. Other interactions, including a divalent sulfur and *π* system, were also observed between the thienopyrimidine moiety and residue Trp780.

### Conformational Flexibility of Ser773 and Ser774 in the p110α–YXY-4F Complex Crystal Structure

**Figure 4:**
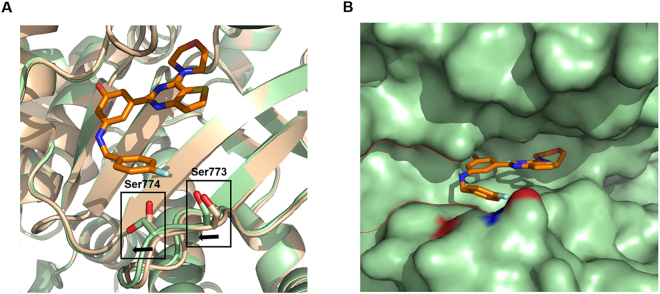
Comparison of *apo* p110α and YXY-4F-p110α complexes. (**A**) Overlay *apo* p110α and YXY-4F-p110α complexes. Apo p110α is colored in wheat and YXY-4F-p110α is colored in palegreen. (**B**) Interface between YXY-4F (orange) and p110α (palegreen). Red curve indicates potential cavity of p110α induced by the binding of YXY-4F.

As shown in Fig. 4, the conformation of YXY-4F with p110α was similar to that of the p110α–PI103 complex^36^. A comparison of the p110α–YXY-4F and *apo* p110α complexes (PDB ID: 4L1B) suggested that a pronounced conformational change was associated with Ser773 and Ser774. The Ser774 side chain moved away from the ATP catalytic site in the YXY-4F-bound-p110α complex (Fig. 4A), which produced ample space to accommodate the phenyl groups linked to the NH_2_ group in compound YXY-4F. Ser773 also moved to accommodate the distance between the phenyl fluorine and the Ser773 hydroxyl group to form a hydrogen bond. Collectively, these data suggested a significant shift in the conformation of Ser773 and Ser774 when compound YXY-4F was bound to PI3Kα, which indicated that the p110α residues Ser773 and Ser774 in the ATP binding site induced the pocket to adopt a different shape after YXY-4F binding (Fig. 4B).

Comparison with our previous PI3K–9d crystal structure showed that compounds 9d and YXY-4F both bound to the ATP-binding pocket on the p110α kinase catalytic domain with similar backbone conformations. While compound 9d induced additional space in the catalytic site by changing the conformation of the p110α Lys802 side chain, compound YXY-4F led to a significant shift in the conformation of Ser773 and Ser774 when bound to p110α (Supplementary Table 1). Additionally, the new hydrogen bond formed in the PI3K–YXY-4F complex and flexible conformation of the fluoro-benzyl moiety might be detrimental to the binding affinity.

**Figure 5:**
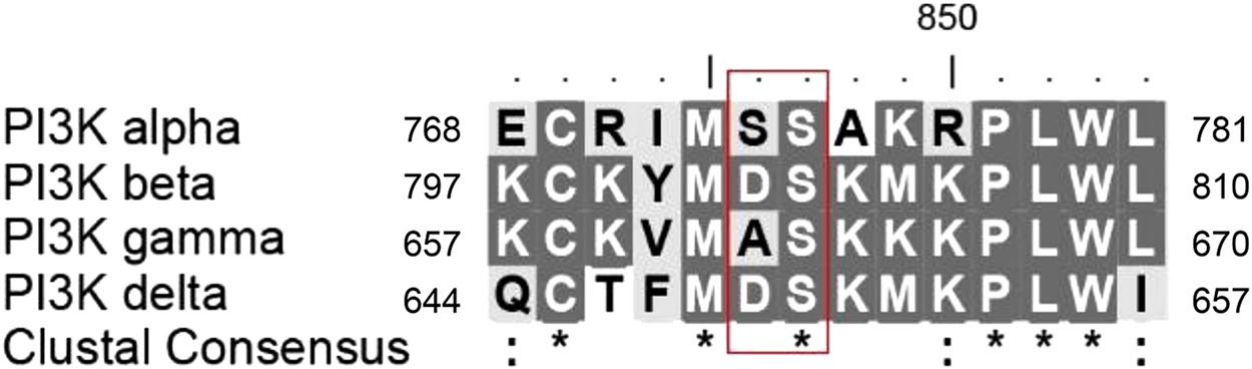
Binding site alignment of Class I PI3K isoforms α, β, γ, δ.

After comparing PI3Kα with other PI3K isoforms, we found that the 773(S) residue in PI3Kα was quite different from those in PI3Kβ (D), γ (A), and δ (D), while the Lys802 residue was conserved (Fig. 5 and Supplementary Fig. 1). The inducible pocket in the PI3Kα-YXY-4F complex, which was different from that of the other PI3K isoforms, might provide a potential new avenue to facilitate further modification of YXY-4F to improve its selectivity and efficacy.

## Discussion

A number of diverse structural PI3Kα inhibitors have been reported^22^. However, their ADMET profiles have limited their therapeutic potential, and drug resistance is also a major obstacle. Drug resistance is mainly caused by hot spot mutations, which may be able to be overcome by developing high affinity inhibitors for the novel PI3Kα binding pocket.

We discovered a moderate PI3Kα inhibitor YXY-4F and obtained the unique crystal structure of YXY-4F in complex with PI3Kα. Although YXY-4F has limited efficacy compared to the successful idelalisib, the compound’s lower molecular weight and previously uncharacterized induced subpocket in the PI3Kα substrate site provide valuable directions to improve the selectivity and potency of these PI3Kα inhibitors^5,26^.

Based on the crystal structure, we will focus on the further optimization of YXY-4F in the following two directions. First, it has been suggested that the hydroxyl group on the YXY-4F phenyl ring might lead to its poor pharmacokinetic profile, including low metabolic stability or solubility. Thus, we can explore the use of an indazole heterocycle as a replacement for phenol to improve multiple aspects of YXY-4F, including its physicochemical properties, metabolic stability, and potency, based on the bioisosterism principle^37–41^. Second, PI3Kα–YXY-4F induced additional space due to a conformational change in Ser773 and Ser774 in the PI3Kα ATP catalytic site (Fig. 4B), which can guide further optimization of the substituents around the NH group and phenyl group to improve the selectivity and potency of YXY-4F for PI3Kα. Meanwhile, the corresponding biomarkers that monitor the drug toxicity of our potent PI3Kα inhibitors and allow for a safety assessment as well as evaluate drug activity to show pharmacological effects will guide further optimization of the ADMET profiles^5^.

In summary, the new PI3Kα inhibitor YXY-4F and its unique complex crystal structure will provide further insights for PI3Kα inhibitor design. The PI3Kα–YXY-4F co-complex can be used as a starting point for subsequent studies to explore the efficacy of inhibitors via structure-based virtual screening and to propose or prioritize biological assays in our experimental design of future inhibitors.

## Methods

### Expression, purification and crystallization of PI3Kα

The full-length human PI3Kα gene template, including human p110α and p85 (Accession No. P42336 and NP_852664), was synthesized at Vivabiotech. A GSPGISGGGGG linker sequence joined the two genes through PCR amplification. To express the N-6*His tag-TEV site-p85α (318–615) - GSPGISGGGGG-full length p110α fusion protein, the joined genes were sub-cloned into the pFastBac HtB vector (Invitrogen). Through sequencing, we verified a positive clone containing the pFastBac-His–TEV-p85α (318–615) - GSPGISGGGGG -p110α vector.

Then, we transformed the FastBac-His–TEV-p85α(318–615)-GSPGISGGGGG–p110α vector into DH10Bac *Escherichia coli* for transposition into the bacmid and selected positive clones on a blue/white LB agar plate. The recombinant bacmid, which was isolated from positive clones, was transfected into SF9 cells to generate recombinant virus stocks, which were amplified for two cycles before infecting SF9 cells at a multiplicity of infection (MOI) of 2. Infection of SF9 cells at a density of 2 × 10^6^ cells/mL with baculovirus stocks at an MOI of 3 was allowed to proceed at 27 °C for 48 hours at 24.5 rocks per minute on an orbital shaker. An approximately 75% average cell viability was observed at the harvest. The cell suspension was frozen and stored at −80 °C until protein purification.

Using two passes through an Avestin pressure drop homogenizer at 12 kpsi, cells were lysed and then centrifuged for 1 h at 15,000 × g. The supernatant was then mixed with Ni–NTA agarose (Qiagen) for 3 hours at 4 °C. We collected the resin and washed it with 100 ml of buffer A **(**250 mM NaCl, 20 mM Tris–HCl pH 7.8, 20 mM imidazole, 2 mM TCEP, and 5% glycerol**)** and 100 ml of buffer B **(**250 mM NaCl, 40 mM imidazole, 20 mM Tris–HCl pH 7.8, 2 mM TCEP, and 5% glycerol**)** before elution with buffer C **(**250 mM NaCl, 250 mM imidazole, 20 mM Tris–HCl pH 7.8, 2 mM TCEP, and 5% glycerol**)**. Buffer A (20 mM Tris-HCl pH 8.0 and 5% glycerol**)** was used to dilute the protein to a ratio of 1:5, and the proteins were purified by ion-exchange chromatography on a Sepharose Q column (eluted by a linear gradient from A to B [buffer A is 20 mM Tris-HCl pH 8.0 and 5% glycerol; buffer B is 20 mM Tris-HCl pH 8.0, 1 M NaCl, and 5% glycerol]). The protein was then concentrated to 5 ml and purified by size-exclusion chromatography on a Superdex 200 column with 20 mM Tris, 2 mM TCEP pH 7.8, 0.2 M NaCl, and 5% glycerol. The resulting protein was almost 95% pure and identified by SDS–PAGE.

The crystallization conditions were screened using 2 μl hanging drops (1 μl protein + 1 μl reservoir) at 18 °C with 11 mg of the His–p85α (318–615)-linker–p110α fusion protein in buffer (200 mM NaCl, 20 mM Tris-HCl, 2 mM TCEP pH 7.8, and 5% glycerol). After screening, a small crystal was identified in solution (0.2 M lithium sulfate, 0.1 M Tris pH 8.5, and PEG 2000 MME). We used hair seeding to obtain a larger crystal in optimized mother liquor (0.1 M Tris pH 8.5, 0.15 M lithium sulfate, and 30% (w/v) PEG 1000 MME). Finally, a diffraction-quality crystal was obtained after repeated rounds of macro-seeding. *Apo* crystals were soaked in 10 mM compound YXY-4F for 2 hours and then mounted according to the above method.

### *In vitro* PI3-Kinase Assays

Compound YXY-4F was dissolved in 100% dimethylsulfoxide (DMSO) at 10 mM. The solution was diluted to the desired concentrations immediately before each experiment. The kinase activities of the purified PI3Ks were determined with the PI3K HTRF Assay (Millipore) according to the manufacturer’s protocol. Briefly, the EC_80_ concentration of each enzyme containing 10 μM PIP2 was incubated in assay buffer on a white 384-well plate (Perkin Elmer). After incubation for 30 min at room temperature, the reaction was initiated by addition of ATP and terminated by addition of the stop solution and detection mix. The final concentration of ATP was 5 μM for p110α. The plate was then sealed and incubated overnight at room temperature. The intensity of the light emission was measured by an EnVision Multilabel Reader (PerkinElmer) in TR-FRET mode (excitation at 320 nm and emission at 665 nm) as previously described^42^. The IC_50_ values^43^ were calculated by fitting the data to a logistic curve using the GraphPad Prism 6 software.

## Electronic supplementary material

Supporting information
