# Supplementary material for: New Insights into PI3K Inhibitor Design using X-ray Structures of PI3Kα Complexed with a Potent Lead Compound
Source: Sci Rep. 2017 Nov 6;7:14572. doi: 10.1038/s41598-017-15260-5 (PMC5674032; doi:10.1038/s41598-017-15260-5)
Supplement: Supplementary file 1 — Supporting information [file 41598_2017_15260_MOESM1_ESM.doc]

**Supporting Information**

**New Insights into PI3K Inhibitor Design using X-ray Structures of PI3Kα Complexed with a Potent Lead Compound**

Xiuyan Yang^1,2,||^, Xi Zhang^3,||^, Min Huang^2,||^, Kun Song^2^, Xuefen Li^3^, Meilang Huang^4^, Linghua Meng^3,*^, Jian Zhang^1,2,*^

^1^ Institute of Bioinformatics and Medical Engineering, School of Electrical and Information Engineering, Jiangsu University of Technology, Changzhou, 213001, China

^2^ Department of Pathophysiology, Chemical Biology Division of Shanghai Universities E-Institutes, Key Laboratory of Cell Differentiation and Apoptosis of Chinese Ministry of Education, Shanghai JiaoTong University, School of Medicine, Shanghai 200025, China

^3^ Division of Anti-tumor Pharmacology, State Key Laboratory of Drug Research, Shanghai Institute of Materia Medica, Chinese Academy of Sciences, Shanghai 201203, China

^4^ School of Chemistry and Chemical Engineering, Queen’s University Belfast, Northern Ireland, United Kingdom

* Correspondence to: Jian Zhang (Phone: +86-21-63846590-776922; Fax: +86-21-64154900; Email: [jian.zhang@sjtu.edu.cn](mailto:jian.zhang@sjtu.edu.cn)) or Linghua Meng (Email: lhmeng@simm.ac.cn)

*^||^* X. Yang, X. Zhang, and M. Huang contributed equally to this work.

**Content Page number**

**Scheme 1. Synthesis of compounds YXY-4F S3**

**Synthesize protocols for YXY-4F S4-S7**

**Table S1 S8**

**Figure S1 S9-S10**

**Scheme 1. Synthesis of compounds YXY-4F**

**Synthesis of compounds YXY-4F:**

**Step a** Thieno[3,2-d]pyrimidine-2,4(1H,3H)-dione (Compound **1**): A mixture of methyl 3-aminothiophene-2-carboxylate (90.0 g, 573 mmol, 1.0 eq) and urea (277.6 g, 4.6 mol, 8.0 eq) was heated at 190°C for 3-4 h and cooled to room temperature. To the reaction mixture was added aq. NaOH (10%, 800 mL). After stirring at ambient temperature for 1 h, the solid was removed by filtration. The filtrate was acidified with HCl to pH 3-4, the precipitated solid was collected by filtration, washed with water and dried in vacuo. White solid (87 g, 89%). m.p.:280-285°C. MS (m/z): 169.0 [M+1]^+^. ^1^H NMR (400 MHz, DMSO-d_6_): δ 6.92 (d, J = 5.2 Hz, 1H), 8.05 (d, J = 5.2 Hz, 1H), 11.0-11.5 (br, 2H).

**Step b** 2,4-Dichlorothieno[3,2-d]pyrimidine (Compound **2**): Phosphorous oxychloride (152 mL, 1.67 mol, 7.0 eq) was added slowly to cold solution of compound **1** (40 g, 238 mmol, 1.0 eq) and N,N-dimethylaniline (22.5 mL, 179 mmol, 0.75 eq) in acetonitrile (250 mL) while maintaining the temperature below 20°C. The mixture was then heated to 85°C and stirred for 24 h. The reaction mixture was cooled to 15°C, and then poured slowly onto a mixture of ice and cold water (360 mL). The resulting slurry was filtered, rinsed with cold water (200 mL). The cake was dried in vacuum oven at 40°C for 24 h. Yield (40.5 g, 83%) as white solid. M.p.:245-250°C. MS (m/z): 205.0 [M+1]^+^.^1^H NMR (400 MHz, DMSO-d_6_): δ 7.75 (d, J = 5.2 Hz, 1H), 8.71 (d, J = 5.2 Hz, 1H).

**Step c** 4-(2-Chlorothieno[3,2-d]pyrimidin-4-yl)morpholine (Compound **3**): A mixture of compound **2** (8.68 g, 42.34 mmol) and morpholine (8.11 mL, 93.15 mmol) in methanol (150 mL) was stirred at room temperature for 1 h. The reaction mixture was then filtered, washed with water (50 mL x 3) and methanol (50 mL x 1) to give the title compound **3**. Yield 11.04 g, 100%, white solid. MS: 257[M+1]^+^; ^1^H NMR (400 MHz, DMSO-d_6_): δ 3.76 (t, J= 4.8 Hz, 4H), 3.91 (t, J= 4.8 Hz, 4H), 7.41(d, J= 5.6 Hz, 1H), 8.31 (d,J= 5.6 Hz, 1H).

**Step d** 3-nitro-5-(4,4,5,5-tetramethyl-1,3,2-dioxaborolan-2-yl)phenol (Compound **4**): 3-bromo-5-nitrophenol (1.09g, 5.0 mmol), bis (tetra methyl ethylenedioxy) diborane (1.4g, 5.5 mmol) and Potassium carbonate (2.07g, 15 mmol) were dissolved in 1,4-diethylene dioxide 30 mL. In addition to the solution 1,1'-Bis(diphenylphosphino) -ferrocene]dichloropalladium(II) (0.12g, 0.16 mmol) was adding heated and stirring for 5 hours at 120 °C under a nitrogen atmosphere. After cooling, catalyst was separated by filtration and it was washed twice with 1,4-diethylene dioxide 10 mL. Filtrate was concentrated under reduced pressure. The residue was purified by silica gel column chromatography to obtained Compound **4** 1.02g (yield 77%) as white solid. MS: 266 [M+1]^+^; ^1^H NMR (400 MHz, DMSO-d_6_): δ 1.31 (s, 12H), 6.10 (s,1H), 7.33 (d, 1H), 7.37 (d, 1H), 7.66 (s, 1H).

**Step e -(4-morpholinothieno[3,2-d]pyrimidin-2-yl)-5-nitrophenol** (Compound **5**): Compound **3** (1.28g, 5.0 mmol), Compound 4 (1.32g, 5.0 mmol), Potassium carbonate (2.07g, 15 mmol) were dissolved in 1,4-diethylene dioxide 30 mL. Then trakis(triphenylphosphine)palladium (0.18g, 0.16 mmol) was added under a nitrogen atmosphere. The mixture was stirred and heated at 120 °C for 8 hours. After the reaction the mixture was stirred, catalyst was separated by filtration and it was washed twice with 1,4-diethylene dioxide 10 mL. Filtrate was concentrated under reduced pressure. The residue was purified by silica gel column chromatography to obtained Compound **5** 1.07g (yield 60%) as white solid. MS: 359 [M+1]^+^;  ^1^H NMR (400 MHz, DMSO-d_6_): 3.92 (t, 4H), 4.00 (t, 4H), 6.10 (s, 1H), 7.10 (s, 1H), 7.20 (s, 1H), 7.47 (d, *J= 12 Hz*, 1H), 8.24 (d, *J= 12 Hz*, 1H), 9.04 (s, 1H).

**Step f 3-**amino-5-(4-morpholinothieno[3,2-d]pyrimidin-2-yl)phenol (Compound **6**): To a solution of Compound **5** (77mg, 0.216mmol) in a mixture in 10mL of 95% ethanol, ammonium chloride (35mg, 0.648mmol) and iron powder (121mg, 2.16mmol) were added. The reaction was refluxed for 4 hours, then cooled at room temperature and filtered on Celite washing with ethanol. The solvent was evaporated under vacuum, and the residue was dissolved in ethyl acetate and washed with saturated aq. NaHCO_3_, water and brine. The organic phase was dried over sodium sulfate, evaporated and purified by column chromatography on silica gel (ether: ethyl acetate=2:1) to obtain Compound **6** 63.7mg (yield 90%) as yellow solid. MS: 329 [M+1]^+^;  ^1^H NMR (400 MHz, DMSO-d_6_): 3.92 (t, 4H), 4.00 (t, 4H), 5.25 (s, 2H),6.10 (s, 1H), 7.10 (s, 1H), 7.20 (s, 1H), 7.47 (d, *J= 12 Hz*, 1H), 8.24 (d, *J= 12 Hz*, 1H), 9.04 (s, 1H).

**Step g** 3-((4-fluorobenzyl)amino)-5-(4-morpholinothieno [3,2-d] pyrimidin-2-yl)phenol (Compound **YXY-4F**): Compound **6** (49mg,0.15mmol) in tetrahydrofuran (10 mL) was treated with 4-fluoro benzaldehyde (16mg, 0.15 mmol) and sodium triacetoxyborohydride (317mg, 1.5mmol). After 3 h, the mixture was partitioned between H_2_O (20 mL) and EA (20 mL), the organic layer collected and the aqueous phase extracted with EA (3x10mL). The extracts were combined with the solvent layer and washed with H_2_O (20 mL). The solvents were then removed and the residue was chromatographed to give the derivative **YXY-4F** 50mg (yield 80%) as yellow solid. MS: 437 [M+1]^+^;  ^1^H NMR (400 MHz, DMSO-d_6_): 3.80 (t, 4H), 3.95 (t, 4H), 4.27 (d, 2H), 5.25 (d, 2H), 6.08 (s, 1H), 6.35 (t, 1H), 7.12 (m, 2H), 7.18 (dd, 2H), 7.41 (dd, 2H), 7.47 (d, *J= 12 Hz*, 1H), 8.23 (d, *J= 12 Hz*, 1H), 9.06 (s, 1H); ^13^C NMR (400 MHz, DMSO-d_6_) 46.2, 46.4, 66.4, 101.5, 103.8, 104.8, 112.3, 115.3, 115.6, 125.3, 129.2, 129.3, 134.1, 137.1, 137.2, 139.9, 158.1, 158.5, 160.1, 162.6, 162.8.

**Supplementary Table S1 Comparison of Crystal Structure PI3Kα–9d and PI3Kα–YXY-4F Complex**

| Crystal Complex | PDB ID | Binding site | Induced residues | PI3KαIC_50_ (μM) |
| --- | --- | --- | --- | --- |
| PI3Kα/YXY-4F | 5XGH | ATP | Ser 773& 774 | 1.02 +0.005 |
| PI3Kα/9d | 4L2Y | ATP | Lys802 | 0.0206 ± 0.001 |

**
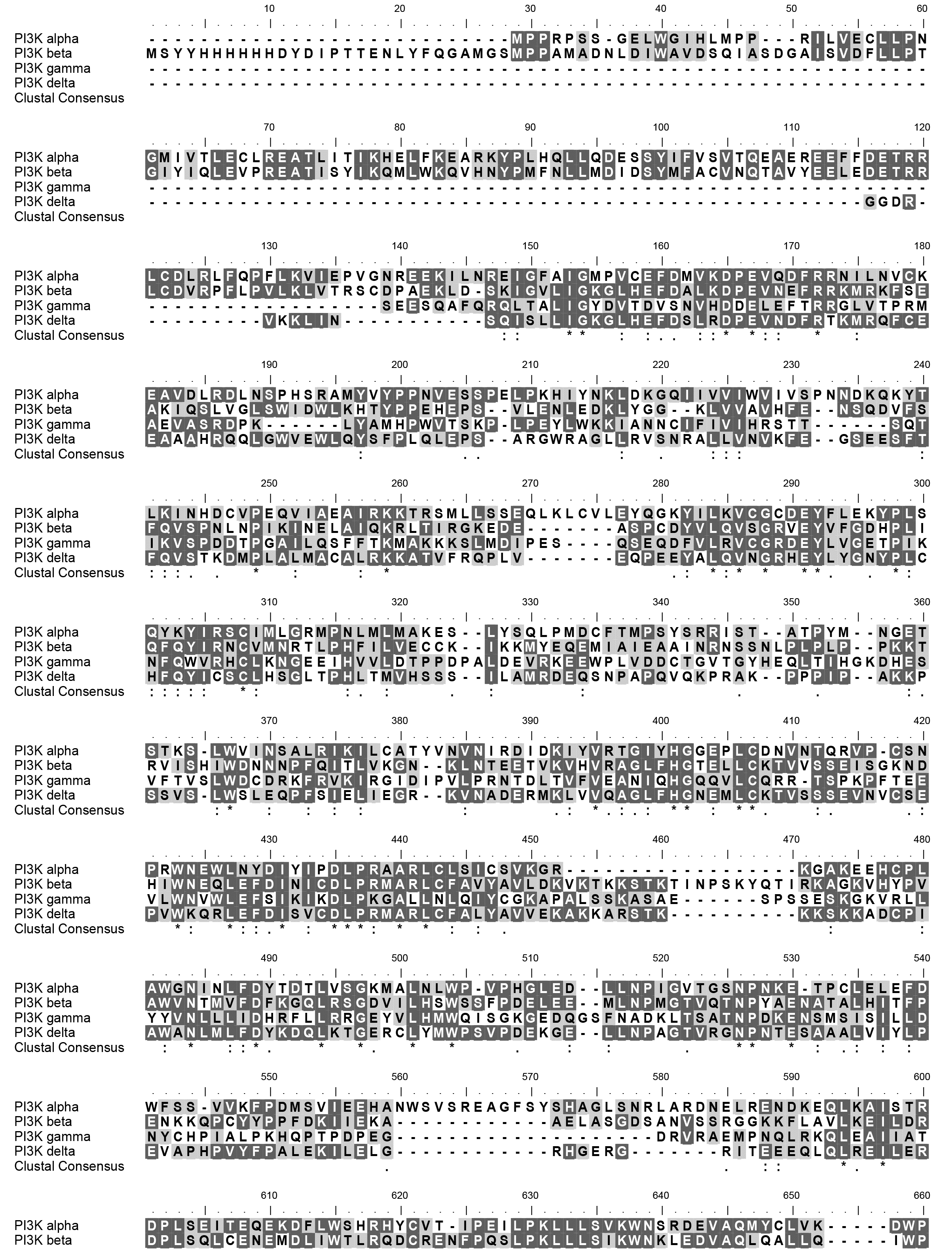
**


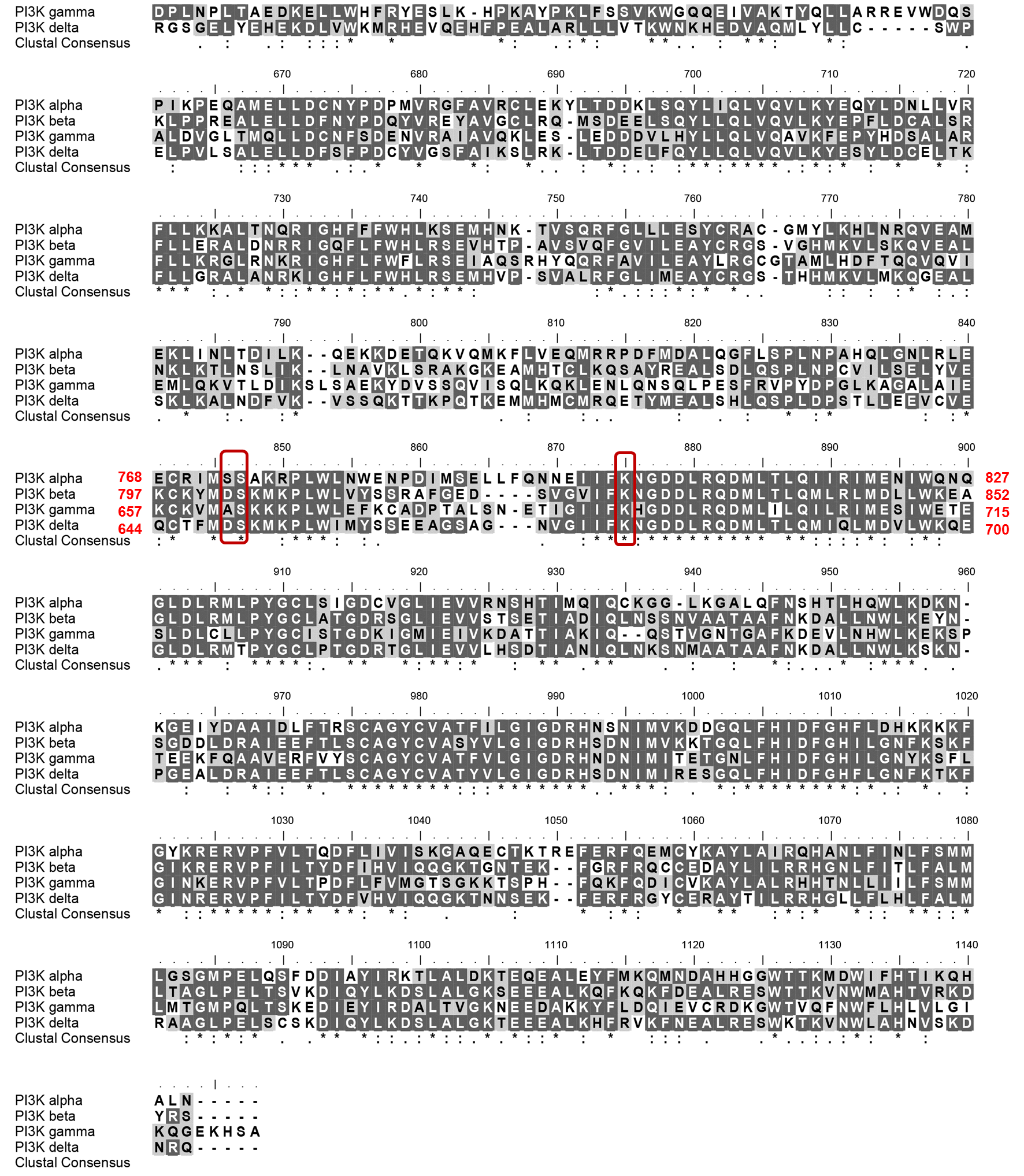


**Supplementary Figure 1**. **Alignment of Class I PI3K isoforms α, β, γ, δ.**
